# Urinary Excretion of 6-Sulfatoxymelatonin, the Main Metabolite of Melatonin, and Mortality in Stable Outpatient Renal Transplant Recipients

**DOI:** 10.3390/jcm9020525

**Published:** 2020-02-14

**Authors:** Anna van der Veen, Isidor Minović, Martijn van Faassen, Antόnio W. Gomes-Neto, Stefan P. Berger, Stephan J. L. Bakker, Ido P. Kema

**Affiliations:** 1 Department of Laboratory Medicine, University of Groningen, University Medical Center Groningen, 9713 GZ Groningen, The Netherlands; i.minovic@umcg.nl (I.M.); h.j.r.van.faassen@umcg.nl (M.v.F.); i.p.kema@umcg.nl (I.P.K.); 2 Department of Internal Medicine, University of Groningen, University Medical Center Groningen, 9713 GZ Groningen, the Netherlands; a.w.gomes.neto@umcg.nl (A.W.G.-N.); s.p.berger@umcg.nl (S.P.B.); s.j.l.bakker@umcg.nl (S.J.L.B.)

**Keywords:** 6-Sulfatoxymelatonin, Melatonin, Kidney Transplantation, Urinary Excretion, LC-MS/MS

## Abstract

Melatonin is a multifaceted hormone which rises upon the onset of darkness. Pineal synthesis of melatonin is known to be disturbed in patients with end-stage renal disease, but it is not known if its production is restored to normal after successful renal transplantation. We hypothesized that urinary excretion of 6-sulfatoxymelatonin, the major metabolite of melatonin, is lower in renal transplant recipients (RTRs) compared to healthy controls and that this is associated with excess mortality. Urinary 6-sulfatoxymelatonin was measured via LC-MS/MS in 701 stable outpatient RTRs and 285 healthy controls. Median urinary 6-sulfatoxymelatonin in RTR was 13.2 nmol/24 h, which was 47% lower than in healthy controls. Urinary 6-sufatoxymelatonin appeared undetectable in the majority of 36 RTRs with diabetic nephropathy as primary renal disease. Therefore, this subgroup was excluded from further analyses. Of the remaining 665 RTRs, during 5.4 years of follow-up, 110 RTRs died, of whom 38 died due to a cardiovascular cause. In Cox-regression analyses, urinary 6-sulfatoxymelatonin was significantly associated with all-cause mortality (0.60 (0.44–0.81), *p* = 0.001) and cardiovascular mortality (0.49 (0.29–0.84), *p* = 0.009), independent of conventional risk factors and kidney function parameters. Based on these results, evaluation and management of melatonin metabolism could be considered for improvement of long-term outcomes in RTRs.

## 1. Introduction

Many patients with end-stage renal disease (ESRD) experience sleep-related problems. Although renal transplantation is the preferred treatment for end-stage renal failure, the quality of sleep in these patients remains lower compared to the general population [1]. A combination of factors, including chronic treatment with immunosuppressive medication, comorbidities, depression, and stress have been indicated to adversely affect the quality of sleep in renal transplant recipients (RTRs) [2,3]. Importantly, poor sleep quality has been implicated as a contributor to poor long-term outcomes and premature mortality in patients with chronic kidney disease [4,5].

Melatonin is a multifaceted hormone that rises upon the onset of darkness. Melatonin is synthesized from tryptophan and serotonin in the pineal gland. Melatonin present in the circulation plays a vital role in the circadian rhythm and has been associated with the sleep–wake cycle [6,7]. After entering systemic circulation, where melatonin exerts its multiple functions via G protein-coupled melatonin-receptors, melatonin is metabolized by the liver, where approximately 90% of melatonin is hydroxylated and subsequently conjugated to form 6-sulfatoxymelatonin (6-SM), which is excreted in the urine [8,9]. The 24 h urinary 6-SM excretion therefore provides an integrated measurement of melatonin production in circulation over a day [10].

Melatonin production has been shown to be severely disturbed in patients with ESRD [11]. It is not known whether pineal melatonin production is restored to normal after successful renal transplantation. Since poor sleep quality has been associated with poor long-term outcomes and mortality in patients with chronic kidney disease, we hypothesized that low endogenous melatonin production in RTRs is associated with increased risk for mortality in these patients. We therefore aimed to compare 24 h urinary 6-SM excretion between RTRs and healthy controls and to prospectively analyse the association of 24 h urinary 6-SM levels with mortality in a large cohort of stable outpatient RTRs, making use of an existing biobank. In these latter analyses, diabetes, in particular diabetic nephropathy, and the use of beta-blockers were of particular interest, because both have been reported to be associated with low endogenous melatonin synthesis and low urinary 6-SM excretion [12,13,14,15].

## 2. Materials and Methods

### 2.1. Study Population

For this study, we performed post-hoc analysis on an existing biobank underlying this prospective cohort study. The cohort comprised RTRs with a graft that had been stable for at least one year. The RTRs visited the outpatient clinic of the University Medical Center Groningen (UMCG), the Netherlands, between November 2008 and June 2010, as described previously [16]. Out of the 817 eligible RTRs, 707 were included and written informed consents were obtained. The median time after transplantation was 5.5 (interquartile range (IQR) 2.0–12.1) years (transplant vintage). For 701 out of the 707 RTRs, 24 h urine samples were available for 6-SM analysis. As control group, 285 healthy controls were included. These controls were kidney donors who participated in a screening program and were included in another cohort. None of the donors had a history of kidney disease, diabetes, or cardiovascular disease. The Medical Ethical Committee of the UMCG approved the study protocol (METc 2008/186) according to the principals of the Declaration of Helsinki.

### 2.2. Data and sample collection

Fasting blood samples of RTRs were drawn in the morning on the day following collection of 24 h urine samples. Routine laboratory analysis in plasma and urine were performed shortly after collection, according to standard laboratory procedures. Plasma and urine samples were stored at −80 °C. Detailed information on the laboratory measurements in this RTR-cohort is described elsewhere [17]. The UMCG renal transplantation database provided information such as date of transplantation, primary renal disease, history of acute rejection, dialysis vintage, and donor status. Data on causes of death were obtained from patients’ records. The glomerular filtration rate (eGFR) was estimated using an equation that included both creatinine and cystatin C [18]. The use of medication and vitamin supplementation were recorded according to patients’ medical records.

### 2.3. Urinary 6-SM Laboratory Measurements

6-SM was measured in 24 h urine samples via isotope dilution liquid chromatography (Pursuit XRS Diphenyl column, Agilent, Santa Clara, CA, USA) combined with tandem mass spectrometry (SPE-LC-MS/MS) (Symbiosis™ Pharma system, Spark Holland, Emmen, the Netherlands and XEVO TQS, Waters, Milford, MA, USA). The urine samples were not stabilized and the pH was not altered. 6-SM-d4 (CacheSyn Inc., Mississauga, Canada) was used as the internal standard. The inter-assay coefficient of variation was <5.4%. The lower limit of quantitation (LLOQ) for 6-SM in urine was 0.20 nmol/L. If a urinary 6-SM value was below the LLOQ, the value was set to 0.20 nmol/L to allow for inclusion of these subjects in further analysis.

### 2.4. Clinical Endpoints

The primary endpoint of this study was all-cause mortality related to 24 h urinary 6-SM output in RTRs. A secondary endpoint was cardiovascular mortality. Cardiovascular mortality included death by cerebrovascular disease, ischemic heart disease, heart failure, or sudden cardiac death according to the International Classification of Diseases, 9th revision (ICD-9), codes 410–447. Endpoints were recorded until the end of September 2015 [19].

### 2.5. Statistical Analysis

Statistical analyses were performed with SPSS statistics version 22.0 (IBM, Armonk, NY, USA). The data are presented as mean ± standard deviation (SD), as median (IQR) or as number (percentage, *n*%). A *p*-value < 0.05 was considered statistically significant. Normal distribution was tested with histograms and probability plots. Non-normally distributed data were transformed to ^10^log, in order to meet the assumptions for linear regression analysis.

Differences between RTRs and healthy controls were determined using univariable regression analyses. Since 24 h urinary 6-SM excretion may be confounded by age, sex, and eGFR as an indication for kidney function, additional multivariable regression analyses were performed, expressed as standardized β. Standardized β allows the comparison of variables with different units, since standardized β makes use of the SD. The higher the standardized β, the stronger the effect.

As the presence of diabetic nephropathy as primary renal disease and the use of beta-blockers have been reported to be associated with particularly low urinary 6-SM excretion [12,13,14,15], we separately evaluated whether they were associated with a particularly high frequency of undetectable urinary 6-SM concentrations, which might preclude further analyses on urinary 6-SM excretion in these subgroups.

The RTR population was divided into tertiles of 24 h urinary 6-SM excretion. To identify associations of urinary 6-SM excretion with baseline variables, univariable linear regression analyses were performed (*p*-trend). To identify independent determinants of 24 h urinary 6-SM excretion, multivariable linear regression analyses were performed. Multivariable linear regression models were constructed using backward selection, and included variables that were associated with urinary 6-SM excretion in the univariable analysis with a *p*-value of <0.20. For related variables, the variable with the strongest association was included in the analysis. All variables were entered into the linear regression model. The variable with the highest *p*-value was excluded and the regression analysis was performed again without this variable. This was repeated until all the variables had a *p*-value below the significance level of 0.05.

To test whether urinary 6-SM excretion was associated with all-cause and cardiovascular mortality, the tertiles of urinary 6-SM excretion were analyzed by Kaplan–Meier analysis with log-rank testing. To report the number of RTRs still at risk of an event at the follow-up time-points of 0, 1, 2, 3, 4, 5, and 6 years, “life tables” were run. Subsequently, Cox proportional hazard regression analyses, with adjustment for potential confounders, were performed on the continuous variable of urinary 6-SM excretion, including the tertiles. The first tertile served as the reference group, and the hazard ratio (HR) was therefore set to 1. An HR lower than the reference value of 1 indicated a poorer outcome concerning mortality. Prospective associations were cumulatively adjusted for potential confounders in five models, to avoid overfitting and to keep the number of predictors in proportion to the number of events. Confounders adjusted for included age and sex (Model 2), waist circumference and smoking (Model 3), diabetes and serum albumin (Model 4), and finally beta-blocker use, eGFR, acute rejection, donor status (living or deceased), and proteinuria (Model 5). Proportionality of hazards was tested by examining the Schoenfeld residuals.

## 3. Results

Baseline characteristics of the 701 RTRs and 285 healthy controls are shown in Table 1. Median (IQR) 24 h urinary 6-SM excretion in RTRs was 13.2 nmol/24 h (3.5–31.2 nmol/24 h), which was 47% lower than in the healthy controls (24.9 (11.6–41.2) nmol/24 h). Of the RTR, 81 (12%) had urinary 6-SM concentrations below the detection limits, compared to 3 (1%) in the healthy controls. In multivariable linear regression analyses, the difference in urinary 6-SM excretion between RTRs and healthy controls appeared to be independent of age and sex and partly independent of eGFR.

In the 36 RTRs with diabetic nephropathy as primary renal disease, the frequency of urinary 6-SM below the detection limit was 22 (61%), which was much higher than the frequency of 59 (9%) in the rest of the RTRs. In the 422 RTRs that were using beta-blockers, the frequency of urinary 6-SM below the detection limit was 55 (13%), which was higher than the frequency of 4 (1.6%) in the rest of the RTRs. Because of the very high frequency of urinary 6-SM concentrations below the detection limit, subjects with diabetic nephropathy were excluded from further analyses. Baseline characteristics of the remaining 665 RTRs, presented according to tertiles of urinary 6-SM excretion, are shown in Table 2. RTRs in the highest tertile of urinary 6-SM excretion were significantly younger, had a lower waist circumference, experienced less acute rejection, had higher eGFR levels as a parameter for kidney function, and had a lower prevalence of diabetes, also marked by lower serum glucose and HbA1c levels, and less use of antidiabetic medication. Furthermore, the urinary 6-SM excretion levels were significantly lower in RTRs who had received a kidney from a deceased donor compared to a living donor (10.2 nmol/24 h (IQR 3.2–25.8 nmol/24 h) and 20.5 nmol/24 h (IQR 9.1–39.2 nmol/24 h) respectively, *p* < 0.001, Mann–Whitney U test).

For the assessment of independent associations of urinary 6-SM excretion parameters, multivariable linear regression analysis with backward elimination was performed. It was found that age, history of acute rejection, and beta-blocker use were inversely associated with urinary 6-SM excretion, whereas living donor status was positively associated, as shown in Table 3.

**Figure 1:**
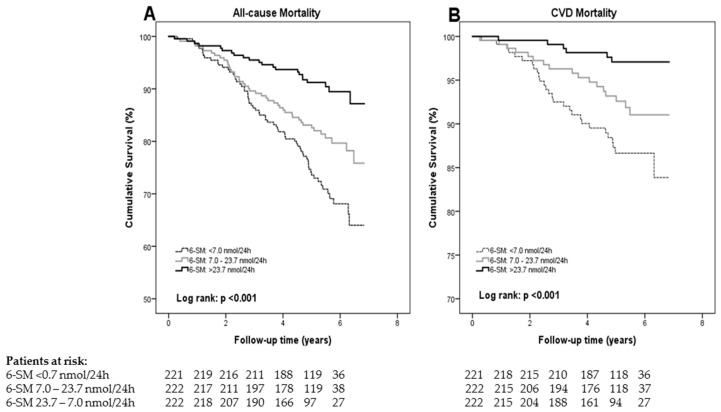
Kaplan–Meier curves for mortality of RTRs according to the tertiles of urinary 6-sulfatoxymelatonin excretion. (**A**) All-cause mortality, and (**B**) cardiovascular mortality. Below the horizontal axis, the numbers of patients still at risk of an event at the follow-up time-points 0, 1, 2, 3, 4, 5, and 6 years are shown. Abbreviations: 6-SM: 6-sulfatoxymelatonin, CVD: cardiovascular disease.

After a median follow-up of 5.4 (4.8–6.1) years, 110 out of 665 RTRs had died (17%), of whom 38 (35%) were due to a cardiovascular cause, including cerebrovascular disease, ischemic heart disease, heart failure, and sudden cardiac death. Kaplan–Meier analysis showed increased risk of all-cause mortality with decreasing tertiles of urinary 6-SM excretion, as shown in Figure 1. Similar results were observed for cardiovascular mortality, both with log-rank *p* < 0.001.

Cox regression analyses revealed that urinary 6-SM excretion, as a continuous variable, was significantly inversely associated with all-cause mortality (HR (95% confidence interval (CI)) = 0.60 (0.44–0.81), *p* = 0.001), independent of potential confounders, including age, sex, waist circumference, smoking, diabetes, serum albumin, eGFR, acute rejection, proteinuria, donor status, and beta-blocker use, as shown in Table 4. Analysis according to the tertiles of urinary 6-SM excretion revealed that RTRs in the lowest tertile (i.e., lowest urinary 6-SM levels) had a higher risk of mortality, compared to RTRs in the intermediate (HR (95% CI) = 0.80 (0.51–1.26)) and highest tertiles (HR (95% CI) = 0.43 (0.24–0.77)).

Cox regression analysis of urinary 6-SM excretion with cardiovascular mortality also displayed a significant association, independent of the potential confounders, (HR (95% CI) = 0.49 (0.29–0.84), *p* = 0.009). Analysis of the tertiles of urinary 6-SM excretion demonstrated an increased cardiovascular-related mortality risk of RTRs in the lowest tertile compared to the middle tertile (HR (95% CI) = 0.70 (0.33–1.51)) and highest tertile (HR (95% CI) = 0.27 (0.09–0.78)), in line with what was found for all-cause mortality.

## 4. Discussion

In this study, we found that 24 h urinary 6-SM excretion, measured with a reliable isotope dilution LC-MS/MS method, was much lower in stable outpatient RTRs than in healthy controls and that low urinary 6-SM excretion was significantly and independently associated with increased risk of premature mortality, particularly cardiovascular mortality.

The role of melatonin during (end-stage) kidney disease and transplantation is increasingly being studied [3,11]. In the case of renal transplantation, a study by Russcher et al. showed that melatonin levels measured in saliva did not improve 3 months after kidney transplantation, and concluded that the time-span after transplantation was too short to see a possible improvement [20]. Burkhalter et al. also measured saliva melatonin in 29 stable RTRs more than 1 year post-transplantation. In these RTRs with sleep–wake disturbances, they found low melatonin levels, mainly in the RTRs without dim-light melatonin onset, and this was related to health impairment in this group [21]. In line with our study results, they found a high percentage of RTRs with fairly low to no melatonin production. These studies showed that restoration of melatonin levels does not occur shortly after transplantation, and our study showed that after a median of 5.5 years following transplantation, the levels are still clearly reduced compared to healthy controls.

Lower urinary 6-SM levels in RTRs compared to healthy controls seem to be partly explained by kidney function, as shown by multivariable linear regression analyses. Besides decreased kidney function, several factors could result in reduced urinary 6-SM in the RTR population. One factor known to reduce melatonin levels is pineal calcification, relatively common with increasing age [22]. A study by Kunz et al. showed reduced urinary 6-SM excretion with increasing pineal calcification [23]. As expected, our RTRs had clearly increased levels of parathyroid hormone (PTH). PTH results in increased mobilization of calcium from bones and promotes reabsorption of calcium in the kidneys [24]. Combined with the reduced renal function in RTRs, less calcium is excreted via the urine; it can then precipitate in soft tissue where it can cause vascular calcification [25,26]. It could be possible that the pineal gland is affected by the calcium deposit. However not much is known yet about the link between PTH and pineal calcification. Another important, extrinsic factor that could influence melatonin levels is the use of medication, and in particular beta-blockers [12,27]. Beta-blockers reduce melatonin production by blocking the adrenergic beta-1 receptor [12]. Inhibition of this receptor prevents the activation of serotonin N-acetyltransferase, necessary for the conversion of serotonin to melatonin. Of the RTRs in this study, 63% used beta-blockers to control hypertension. The cross-sectional data of RTRs on beta-blockers showed significantly lower levels of urinary 6-SM, however, correction for beta-blockers as a potential confounder in prospective analysis did not influence the association of urinary 6-SM excretion with mortality. Additionally, in our cohort of stable RTRs, it was found that patients who had received a kidney from a living donor had higher urinary 6-SM levels compared to patients that had received a kidney from a deceased donor. The waiting time for a kidney from a living donor is usually shorter, generally resulting in considerably less advanced kidney disease and less time spent on dialysis or preemptive transplantation [28,29]. In line with this, we found a strongly significant association between higher cold ischemia time and lower urinary 6-SM excretion. Lastly, multiple studies have demonstrated that RTRs experience poor sleep quality, and a high prevalence of insomnia is found in this population [1,30,31,32]. Melatonin secretion is an important marker for the circadian rhythm and it signals the “biological night”, causing the onset of sleep in humans [8]. The reduced levels of melatonin measured in the RTRs in this study could be influenced by or cause the sleep-related problems. This was recognized by the study of Russcher et al. mentioned earlier, where the authors did not find improved sleep quality and melatonin levels after renal transplantation, despite improved renal function [20].

We found that RTRs with diabetic nephropathy as primary renal disease showed clearly reduced to no detectable urinary 6-SM levels. Diabetes and diabetic complications, such as diabetic nephropathy, are associated with the increased presence of oxidative stress after a persistent period of poor glucose control [33,34]. Patients with diabetic nephropathy often experience serial problems affecting the eyes, arteries, and peripheral nerves. Currently, transplantation is considered the preferred treatment for patients with ESRD caused by diabetic nephropathy [35]. Why the RTRs with diabetic nephropathy as primary renal disease have lower 6-SM compared to the total RTR population needs to be further investigated. Potential mechanisms could include imbalance in the production of reactive oxygen species, increased vascular calcification, retinopathy, or impaired glucose control. Systemic melatonin synthesis is dependent on the light–dark signals transmitted via the retino-hypothalamic tract [8]. These signals might be absent in patients with severe retinopathy, possibly influencing their melatonin levels [36,37]. One study showed that rats with diabetic nephropathy benefit from treatment with melatonin, most likely via reduction of the oxidative stress, demonstrating a potentially important of role melatonin in the treatment of RTRs with diabetic nephropathy as primary renal disease [38].

The present study showed that reduced urinary 6-SM excretion was associated with an increased risk of all-cause mortality in RTRs independent of age, kidney function, and beta-blocker use. In addition to all-cause mortality, this study showed that low urinary 6-SM levels were independently associated with cardiovascular mortality. Cardiovascular disease is a major cause of death in RTRs, caused by conventional risk factors such as hypertension, diabetes, and renal dysfunction [39]. Reduced urinary 6-SM excretion has been linked to coronary artery disease (CAD); the severity of CAD was negatively associated with urinary 6-SM excretion [40]. Melatonin is known to influence the cardiovascular system via both its antioxidant and anti-inflammatory activities [41]. In rats with renovascular hypertension, the cardiac function improved after treatment with melatonin, implying a possible therapeutic role of melatonin [42]. That melatonin could play a possible important role in kidney transplantation as a therapeutic approach was also demonstrated in a study by Li et al. [43]. In this study, with rats, it was shown that donor pre-conditioning with melatonin protected the kidney graft, likely through its antioxidative properties, improving the survival.

Future investigations should focus on whether supplementation of melatonin in RTRs can prevent the known circadian rhythm sleeping disorders and act as a possible cardiovascular protector by lowering the oxidative stress. In line with this, it would be interesting to examine whether melatonin supplementation affects the mortality rates in RTRs by means of a double-blind randomized control trial.

Some limitations of the study should be noted. The 24 h urine samples were collected just once, and the conclusions are therefore based on a single measurement. Since the patients were carefully instructed on how to collect the urine, over- and under-collections were unlikely, as previously tested by sensitivity analysis in the same cohort [44]. An additional limitation of this study was the lack of information on sleep reported by or monitored in the RTRs. Subsequently, there were no data available on the use of sleep-related medication or the presence of sleeping disorders such as obstructive sleep apnea syndrome. Even though many studies have shown a link between melatonin and sleep disturbances, we were not able to examine this in our study, and this would a valuable addition for future studies. Additionally, it would have been interesting to examine the association with mortality in healthy individuals, with sufficient follow-up time to underline the findings in the present study. This study could, for instance, be combined with individuals with and without sleep deprivation, to emphasize the results of the current study possibly even more.

One of the major strengths of this study was the use of 24 h urine for 6-SM analysis, instead of using plasma melatonin or saliva melatonin. Collection of 24 h urine provides insight over a whole day, rather than at a single time-point, as is the case with plasma melatonin or saliva melatonin. This is particularly of interest because melatonin production is influenced by the circadian rhythm. Furthermore, it has been shown that urinary melatonin levels are stable over time within an individual [45]. However, it is important to realize that melatonin levels can be influenced to a certain degree by seasonal changes, working night shifts, and the degree of exposure to artificial light [46,47]. Another strength of this study was the use of LC-MS/MS to measure 6-SM. Compared to immunoassays, LC-MS/MS is more specific, robust, and able to measure low concentrations of urinary 6-SM. Finally, this was an observational study, and it is therefore possible that unmeasured or additional confounding variables might have been present, despite the many potential confounders that we did adjust for.

## 5. Conclusions

In this biobank study, we found significantly lower urinary 6-SM levels in a large group of stable RTRs compared to healthy controls. More importantly, low urinary 6-SM levels in RTRs were independently associated with higher mortality risk, independent of potential confounders. Based on these results, evaluation and management of melatonin metabolism could be considered for improvement of long-term outcome in RTRs.

## Figures and Tables

**Table 1:** Baseline characteristics comparison between healthy controls and RTR^1.^

			Standardized ß		
	Healthy Controls *n* = 285	RTR *n* = 701	Model 1	Model 2	Model 3
6-SM (nmol/24 h)	24.9 (11.6–41.2)^1^	13.2 (3.5–31.2)	−0.244 ***	−0.276 ***	−0.062 **
6-SM (nmol/L)	9.9 (4.5–17.9)	5.2 (1.5–12.7)	−0.152 ***	−0.180 ***	−0.071 **
Urine volume (L)	2.5 (2.0–3.0)	2.4 (1.9–2.8)	−0.069 *	−0.064 *	0.000
Undetectable 6-SM levels, *n*%	3 (1)	81 (12)	0.169 ***	0.178 ***	0.024
Age, years	53.3 ± 10.8^2^	53.0 ± 12.7	−0.019		
Male sex, ***n***%	135 (47)	399 (57)	−0.089**		
eGFR, mL/min per 1.73 m^2^	90.9 ± 14.1	45.0 ± 18.7	−0.749 ***	−0.758 ***	
Total cholesterol, nmol/L	5.4 ± 1.1	5.1 ± 1.1	−0.106 **	−0.095 **	−0.064 **
Waist-circumference, cm	91.1 ± 10.1	98.6 ± 14.7	0.216 ***	0.220 ***	0.066 **
hsCRP, mg/L	1.1 (0.6–2.3)	1.6 (0.7–4.6)	0.113 ***	0.133 ***	−0.002
Serum glucose, mmol/L	5.4 ± 0.6	5.7 ± 1.8	0.060	0.056	0.046 *
HbA1c, %	5.6 ± 0.3	6.0 ± 0.8	0.245 ***	0.258 ***	0.135 ***
PTH, pmol/L	3.3 (2.6–4.2)	8.9 (5.9–14.7)	0.552 ***	0.553 ***	0.192 ***
Beta-blocker use, ***n***%	13 (5)	448 (64)	0.506 ***	0.509 ***	0.217 ***

^1^ Differences between groups were analyzed with univariable and multivariable linear regression analyses, for which the standardized *ß*s are shown (* *p* < 0.05, ** *p* < 0.01, *** *p* < 0.001). Model 1 was a crude model, Model 2 was adjusted for age and sex. Model 3 was adjusted for Model 2 and eGFR; ^1^Median (IQR); ^2^Mean ± SD. Abbreviations: 6-SM: 6-sulfatoxymelatonin; eGFR: estimated glomerular filtration rate; HbA1c: glycated hemoglobin; hsCRP: high-sensitivity C-reactive protein; PTH: parathyroid hormone.

**Table 2:** Baseline patient characteristics of the stable RTRs (without diabetic nephropathy) presented as tertiles of urinary 6-sulfatoxymelatonin ^1.^

Characteristics	Overall *n* = 665	Tertile 1 *n* = 221	Tertile 2 *n* = 222	Tertile 3 *n* = 222	*p*-Value
6-SM (nmol/24 h) *	14.3 (4.4–31.6)	<7.0	7.0–23.7	>23.7	<0.001
**Demographics**					
Age, years	52.8 ± 12.9	58.9 ± 9.9	52.4 ± 12.0	47.0 ± 13.7	<0.001
Male sex (*n*%)	376 (57)	119 (54)	143 (64)	114 (51)	0.96
6-SM (nmol/L)	5.9 (1.8–13.1)	0.97 (0.2–1.8)	5.9 (4.0–8.3)	18.3 (12.2–27.9)	<0.001
Urine volume (L)	2.4 (1.9–2.8)	2.4 (1.9–2.8)	2.4 (1.9–2.8)	2.4 (1.9–2.8)	0.97
Undetectable 6-SM levels (*n*%)	59 (9)	59 (27)	0 (0)	0 (0)	<0.001
Current smoker (*n*%)	81 (12)	24 (11)	35 (16)	22 (10)	0.95
BMI (kg/m^2^)	26.6 ± 4.7	27.0 ± 4.6	26.3 ± 4.4	26.5 ± 5.0	0.09
Alcohol intake (g/d)	3.0 (0.04–11.5)	2.02 (0.03–13.2)	3.49 (0.05–12.1)	3.08 (0.05–9.9)	0.55
Waist circumference (cm)	98.3 ± 14.4	99.6 ± 14.4	99.5 ± 14.1	95.8 ± 14.3	0.004
Body surface area (m^2^)	1.94 ± 0.22	1.91 ± 0.21	1.96 ± 0.22	19.4 ± 0.22	0.47
Systolic BP (mmHG)	135.3 ± 17.3	136.8 ± 17.5	134.9 ± 18.3	134.2 ± 16.0	0.16
Diastolic BP (mmHG)	82.3 ± 10.9	81.8 ± 10.0	83.1 ± 12.0	82.3 ± 10.8	0.39
History of CVD (*n*%)	84 (13)	30 (14)	29 (13)	25 (11)	0.44
**Renal transplantation**					
Transplant vintage (years)	5.5 (2.0–12.1)	6.9 (3.2–14.0)	5.4 (1.5–12.3)	4.9 (1.4–10.4)	0.005
Living donor (*n*%)	228 (34)	45 (20)	82 (37)	101 (45)	<0.001
Acute rejection (*n*%)	172 (26)	63 (29)	67 (30)	42 (19)	0.002
Cold ischemia time (hours)	14.5 (2.6–21.0)	16.2 (8.2–22.5)	12.4 (2.6–20.9)	10.7 (2.4–19.6)	<0.001
Primary renal disease (*n*%)					
*Primary glomerular disease*	197 (30)	61 (28)	75 (34)	61 (27)	0.52
*Glomerulonephritis*	52 (8)	20 (9)	12 (5)	20 (9)	0.55
*Tubulointerstitial disease*	84 (13)	22 (10)	24 (11)	38 (17)	0.10
*Polycystic renal disease*	145 (22)	48 (22)	55 (25)	42 (19)	0.56
*Dysplasia and hypoplasia*	28 (4)	9 (4)	7 (3)	12 (5)	0.56
*Renovascular disease*	40 (6)	12 (5)	14 (6)	14 (6)	0.84
*Other or unknown cause*	118 (18)	48 (22)	35 (16)	35 (16)	0.12
**Laboratory measurements**					
eGFR (mL/min per 1.73 m^2^)	45.4 ± 18.8	41.3 ± 19.5	46.8 ± 17.8	46.8 ± 18.3	<0.001
Serum albumin (g/L)	43.1 ± 3.0	42.7± 2.9	43.0 ± 3.0	43.5 ± 2.9	0.001
Proteinuria (*n*%)	136 (20)	50 (23)	45 (20)	41 (18)	0.07
NT-pro BNP (ng/L)	243 (103–594)	434 (182–1120)	113 (105–432)	143 (67–363)	0.04
Total cholesterol (mmol/L)	5.1 ± 1.1	5.3 ± 1.2	5.0 ± 1.1	5.1 ± 1.0	0.05
LDL cholesterol (mmol/L)	3.0 ± 0.93	3.1 ± 1.0	2.9 ± 0.94	3.0 ± 0.86	0.10
HDL cholesterol (mmol/L)	1.3 (1.1–1.6)	1.3 (1.1–1.6)	1.3 (1.0–1.6)	1.3 (1.1–1.7)	0.37
hsCRP (mg/L)	1.6 (0.7–4.6)	2.1 (0.8–5.1)	1.5 (0.6–4.5)	1.5 (0.7–4.5)	0.009
Serum calcium (mmol/L)	2.4 ± 0.15	2.4 ± 0.15	2.4 ± 0.15	2.4 ± 0.15	0.49
PTH (pmol/L)	8.9 (5.8–14.6)	9.5 (6.3–16.5)	8.9 (5.6–15.3)	8.5 (5.5–13.7)	0.29
**Medication**					
Antihypertensiva					
*Beta-blockers* (*n*%)	422 (63)	183 (83)	140 (63)	99 (45)	<0.001
*Calcium antagonists* (*n*%)	159 (24)	61 (28)	53 (24)	45 (20)	0.08
*ACE inhibitors* (*n*%)	218 (33)	66 (30)	80 (36)	72 (32)	0.51
Immunosuppressive therapy					
*Prednisolone* (*n*%)	658 (99)	218 (99)	218 (98)	222 (100)	0.14
*Calcineurin inhibitor* (*n*%)	370 (56)	120 (54)	125 (56)	125 (56)	0.90
*Proliferation inhibitors* (*n*%)	555 (83)	177 (80)	189 (85)	189 (85)	0.49
*mTOR inhibitors* (*n*%)	24 (4)	10 (5)	6 (3)	8 (4)	0.66
Proton pump inhibitors (*n*%)	323 (49)	125 (57)	105 (47)	93 (42)	0.02
**Diabetes parameters**					
Serum glucose (mmol/L)	5.2 (4.8–6.0)	5.3 (4.8–6.0)	5.3 (4.8–6.1)	5.1 (4.7–5.8)	0.03
HbA1c (%)	5.9 ± 0.8	6.0 ± 0.8	5.9 ± 0.8	5.8 ± 0.7	0.02
Current diabetes (*n*%)	146 (22)	62 (28)	47 (21)	37 (17)	0.02
Pre-transplant diabetes (*n*%)	13 (2)	4 (2)	2 (1)	7 (3)	0.35
Antidiabetica (*n*%)	87 (13)	40 (18)	31 (14)	16 (7)	0.002

^1^ Data are presented as mean ± SD, percentage (*n*%) or median (IQR). The *p*-values (*p* for trend) were obtained by linear regression analyses. Abbreviations: 6-SM: 6-sulfatoxymelatonin; ACE: angiotensin-converting enzyme; BMI: body mass index; BP: blood pressure; CVD: cardiovascular disease; eGFR: estimated glomerular filtration rate; HbA1c: glycated hemoglobin; HDL: high-density lipoprotein; hsCRP: high-sensitivity C-reactive protein; LDL: low-density lipoprotein; mTOR: mechanistic target of rapamycin; NT-pro BNP: N-terminal prohormone of brain natriuretic peptide; PTH: parathyroid hormone.

**Table 3:** Univariable and multivariable associations of 6-sulfatoxymelatonin excretion with clinical parameters in 665 RTRs (without diabetic nephropathy) ^1.^

Urinary 6-Sulfatoxymelatonin Excretion				
	Univariable		Multivariable	
	*Standardized ß*	*p-Value*	*Standardized ß*	*p-Value*
**Demographics**				
Age (years)	−0.351	<0.001	−0.282	<0.001
Sex	−0.002	0.96		
Waist circumference (cm)	−0.115	0.004		
SBP (mmHG)	–0.055	0.16		
**Renal transplantation**				
Transplant vintage (years)	−0.110	0.005		
Living donor	0.224	<0.001	0.130	<0.001
Acute rejection	−0.118	0.002	−0.082	0.02
Cold ischemia time, hours	−0.182	<0.001		
Primary renal disease				
*Tubulointerstitial disease*	0.065	0.10		
*Other or unknown cause*	–0.060	0.12		
**Laboratory measurements**				
eGFR (mL/min per 1.73 m^2^)	0.153	<0.001		
Serum albumin (g/L)	0.130	0.001		
Proteinuria (*n*%)	−0.070	0.07		
NT-pro BNP (ng/L)	−0.081	0.04		
Total cholesterol (nmol/L)	−0.077	0.05		
hsCRP (mg/L)	–0.102	0.009		
**Medication**				
Beta-blockers	−0.324	<0.001	−0.264	<0.001
Prednisolone (mg/d)	0.057	0.14		
Proton-pump inhibitors	–0.094	0.02		
**Diabetes parameters**				
Serum glucose (mmol/L)	−0.087	0.03		
HbA1c (%)	−0.093	0.02		
Current diabetes	−0.089	0.02		
Antidiabetica	–0.118	0.002		

^1^ Multivariable linear regression analysis using backward selection (*p*_out_ > 0.05). Abbreviations: CVD: cardiovascular disease; eGFR: estimated glomerular filtration rate; HbA1c: glycated hemoglobin; hsCRP: high-sensitivity C-reactive protein; NT-pro BNP: N-terminal prohormone of brain natriuretic peptide; PTH: parathyroid hormone; SBP: systolic blood pressure.

**Table 4:** Cox proportional hazards regression analysis for the association of 6-sulfatoxymelatonin with all-cause and cardiovascular mortality in RTRs (without diabetic nephropathy) ^1.^

	Tertile 1	Tertile 2		Tertile 3		Overall	
	*HR (95% CI)*	*HR (95% CI)*	*p-Value*	*HR (95% CI)*	*p-Value*	*HR (95% CI)*	*p-Value*
**All-cause mortality ^a^**							
Model 1 ^c^	1.0 (Ref)	0.59 (0.39–0.90)	0.01	0.27 (0.16–0.46)	<0.001	0.45 (0.34–0.59)	<0.001
Model 2 ^d^	1.0 (Ref)	0.81 (0.53–1.24)	0.34	0.45 (0.26–0.78)	0.004	0.60 (0.45–0.80)	0.001
Model 3 ^e^	1.0 (Ref)	0.76 (0.50–1.12)	0.20	0.44 (0.25–0.76)	0.003	0.60 (0.45–0.79)	<0.001
Model 4 ^f^	1.0 (Ref)	0.73 (0.48–1.12)	0.15	0.42 (0.24–0.73)	0.002	0.58 (0.44–0.77)	<0.001
Model 5 ^g^	1.0 (Ref)	0.80 (0.51–1.26)	0.34	0.43 (0.24–0.77)	0.004	0.60 (0.44–0.81)	0.001
**Cardiovascular mortality ^b^**							
Model 1 ^c^	1.0 (Ref)	0.61 (0.30–1.21)	0.16	0.21 (0.08–0.57)	0.002	0.42 (0.27–0.67)	<0.001
Model 2 ^d^	1.0 (Ref)	0.78 (0.38–1.58)	0.48	0.33 (0.12–0.90)	0.03	0.52 (0.32–0.86)	0.01
Model 3 ^e^	1.0 (Ref)	0.69 (0.34–1.41)	0.31	0.32 (0.12–0.88)	0.03	0.52 (0.32–0.86)	0.01
Model 4 ^f^	1.0 (Ref)	0.63 (0.31–1.29)	0.20	0.28 (0.10–0.76)	0.01	0.48 (0.29–0.78)	0.003
Model 5 ^g^	1.0 (Ref)	0.70 (0.33–1.51)	0.37	0.27 (0.09–0.78)	0.01	0.49 (0.29–0.84)	0.009

^1^ Data are presented as hazard ratio (HR) with the 95% CI and *p*-values according to the tertiles of 6-SM and the continuous data. ^a^ N_events_/N_total_ = 110/665; ^b^ N_events_/N_total_ = 38/665; ^c^ Model 1: crude; ^d^ Model 2: adjusted for age, sex; ^e^ Model 3: waist circumference, smoking; ^f^ Model 4: current diabetes, serum albumin; ^g^ Model 5: eGFR, acute rejection, proteinuria, living donor, beta-blocker. Abbreviations: 6-SM: 6-sulfatoxymelatonin; CI: confidence interval; HR: hazard ratio.
